# Evaluating alignment of UK commercial baby food products with the WHO nutrient and promotion profile model

**DOI:** 10.1007/s00431-025-05971-7

**Published:** 2025-01-11

**Authors:** Cigdem Bozkir, Kubra Esin, Diane Threapleton, Janet E. Cade

**Affiliations:** 1 Faculty of Health Sciences, Nutrition and Dietetics Department, Inonu University, Malatya, Türkiye; 2 Nutritional Epidemiology Group, School of Food Science and Nutrition, University of Leeds, Leeds, UK; 3 Faculty of Health Sciences, Nutrition and Dietetics Department, Tokat Gaziosmanpasa University, Tokat, Türkiye

**Keywords:** UK baby food products, Nutritional standards, Food labelling, Early childhood nutrition, Nutrient and promotion profile model

## Abstract

**Purpose:**

The first 1000 days of life are critical for long-term health outcomes, and there is increasing concern about the suitability of commercial food products for infants, toddlers, and children. This study evaluates the compliance of UK commercial baby food products with WHO Nutrient and Promotion Profile Model (NPPM) guidelines.

**Methods:**

Between February and April 2023, data on 469 baby food products marketed for infants and children under 36 months were collected from the online platforms of four major UK supermarkets. Nutritional composition and labelling information were assessed using the NPPM criteria. Quantitative analyses were performed using IBM SPSS, presenting data as means with 95% confidence intervals.

**Results:**

While 75% of products met the minimum energy content criteria, compliance with total sugar content and protein requirements was 59% and 94%, respectively. Overall, only 45% of products adhered to NPPM nutritional standards. Promotional assessments revealed that no products met the requirements for appropriate nutrient, health, or marketing claims. Furthermore, only 5% of products included adequate statements in support of breastfeeding.

**Conclusion:**

This study highlights the need for stricter nutritional and promotional standards in the UK baby food industry to foster healthier early dietary habits. Regulatory measures are essential to align commercial baby food products with WHO recommendations, reducing inappropriate claims and improving nutritional quality.

**Supplementary Information:**

The online version contains supplementary material available at 10.1007/s00431-025-05971-7.

## Introduction

Early nutrition significantly influences lifelong health, with global efforts emphasizing improved dietary patterns for children under five [1, 2]. However, global trends reveal a concerning rise in childhood overweight and obesity, underscoring an imbalance in early dietary patterns [3, 4]. This imbalance has been attributed to the aggressive marketing of unhealthy processed foods targeted at children [5]. In response, there is a growing call for tighter regulation of foods high in fat, sugar, and salt, particularly those aimed at young children [6, 7].

The transition from milk-based diets to solid foods during weaning is a key opportunity to establish lifelong healthy eating habits [8, 9]. Ensuring adequate nutrient intake during this phase is essential for optimal growth and development [10]. he widespread availability of commercially produced foods for infants and young children (FIYC) has raised concerns over their nutritional quality, particularly levels of sugar, salt, and essential nutrients, which could lead to adverse health effects [11–13]. Additionally, certain marketing strategies may encourage inappropriate feeding practices, such as early solid food introduction, counter to recommended breastfeeding guidelines [12, 14].

The World Health Organization (WHO) has highlighted specific concerns about FIYC products, including high sugar levels often due to added fruit purées, age recommendations that promote early introduction of solid foods (e.g., from 4 months), and nutrient deficiencies that do not adequately meet the needs of infants and young children [15]. In the UK, guidelines further advise limiting added sugars and salt in commercial products and recommend that dried fruits be reserved for mealtimes due to potential dental risks. These guidelines emphasize the necessity of nutritionally balanced products that are aligned with developmental needs and local dietary standards. Public Health England, in particular, advocates for the introduction of a variety of vegetables and single-ingredient foods in home-prepared meals for infants, establishing a benchmark for assessing commercial FIYC products in the UK [16]. Amongst guidelines on complementary foods, parents are encouraged to offer home-made baby foods [16] but 58% of UK babies received commercial baby foods between 6 and 12 months [17].

To address the challenges associated with FIYC nutritional quality and marketing, the Nutrient and Promotion Profile Model (NPPM), developed by the WHO Regional Office for Europe, provides a standardized framework for evaluating these products. The NPPM sets nutrient and promotional standards aimed at reducing sugar, salt, and inappropriate ingredients, regulating potentially misleading promotional practices that might conflict with breastfeeding recommendations, such as marketing products for children under six months or emphasizing nutritional superiority [15]. This model matches UK voluntary recommendations around marketing, labelling and nutrient composition of products [16]. The WHO NPPM has been used for product evaluation since the national recommendations do not yet state specific cut-off points for nutrients relating to specific baby food categories. Our evaluation supports alignment of FIYC with national policies that promote nutrition for infants and young children aged 6–36 months.

The NPPM supports broader public health goals of fostering healthy dietary habits, reducing childhood obesity, and improving long-term health outcomes [18]. By assessing nutritional composition and marketing practices, the NPPM enables informed decision-making by regulators and empowers parents and caregivers to make healthier choices for their children. This study aims to evaluate the compliance of FIYC products in the UK with NPPM criteria.

## Materials and methods

### Sampling and data collection

A comprehensive survey was conducted to evaluate commercially available FIYC products in the UK markets.

The data was collected from the online platforms of four prominent supermarket chains: Tesco, Sainsbury’s, Morrisons, and ASDA. The selected supermarkets cover approximately two-thirds of the UK market [19]. Selection of these supermarkets was based on their widespread presence and representation across diverse UK regions. Data collection occurred between February and April 2023.

Data was initially collected by one researcher (CB) from the websites of four major supermarkets, using search terms like “baby food” and “toddler food.” Products intended for children under 36 months were identified by age recommendations on labels and website descriptions, with selected items cross-checked in-store for accuracy. Additional verification of ingredient details was cross-checked using the Ocado and Waitrose websites, leading to the inclusion of an ingredient column in the NPPM template. Two researchers (CB and DT) independently reviewed the dataset to remove duplicates and confirm labelling accuracy, with any discrepancies resolved through discussion and consensus.

### Data analysis

Nutritional composition, labelling information, and manufacturers’ claims were systematically collected from the respective product pages on the supermarkets’ websites. The collected data were organized and tabulated in Microsoft Excel templates from the NPPM website [20] for further analysis. The food products were categorized into eight main groups based on the website instructions “Cereals,” “Dairy,” “Fruit and vegetables,” “Meals and meal components,” “Snacks and finger foods,” “Ingredients,” “Confectionery,” and “Drinks.”

The data, including nutritional composition parameters and labelling information, was analysed using the NPPM website and IBM SPSS version 29. Quantitative data are presented as means and Confidence Intervals (CI) with nutrient content expressed per 100 kcal and per 100 g. A one-way ANOVA test was used to compare energy density and nutrient content per 100 g across different categories. Labelling and packaging information are presented as frequencies and numbers.

### Nutritional composition

The nutritional composition analysis focused on key parameters including energy, sugar, fat, protein, and salt content of the FIYC products. Evaluation of these nutritional components was carried out in accordance with guidelines outlined by the WHO NPPM for infant and young child feeding. These guidelines provided benchmark values and recommended ranges for optimal nutrition in FIYC [15].

The NPPM guidelines outline specific nutritional requirements:

Energy: Dry cereals/starches must have ≥80 kcal/100 g (prepared), non-dry products (dairy, fruit-based, meals) ≥60 kcal/100 g, and snacks ≤50 kcal per serving.

Protein: Dry cereals with milk ≤5.5 g/100 kcal; savoury meals ≥3 g/100 kcal, with higher levels for meals containing meat, poultry, or fish.

Fat: ≤4.5 g/100 kcal for most products; meals with traditional protein sources may have up to 6 g/100 kcal.

Sugar: Meals and non-fruit snacks should contain ≤15% of energy from total sugars. Added sugars and sweeteners (e.g., syrups, juices) are prohibited (see supplementary file for sugar classification details. In brief, sugar is defined as including any intrinsic sugars contained within plant cell walls, liberated sugars, free sugars, and sugars naturally present in milk (largely lactose)).

Salt: Maximum 0.125 g/100 kcal; products with cheese can contain up to 0.25 g/100 kcal [15] (Supplementary file Table 1).

### Labelling information assessment

The labelling information of FIYC products was scrutinized for compliance with NPPM specifications. Key aspects analysed were ingredient list completeness (i.e. reporting proportions of major and fruit ingredients), nutritional claims (e.g., “no added sugar” or “low in sugar”), health and marketing claims (e.g., “organic food,” “source of vitamins or minerals,” “supports healthy growth,” “tasty/yummy/delicious”), the presences of a statement to support and protect breastfeeding, recommendations for children not to drink pureed foods via a spout, and suitable preparation instructions provided by manufacturers (Supplementary file Table 2). Additionally, the NPPM recommends front-of-pack flags to alert consumers to the presence of high sugar contents. Fruit/vegetable products or cereals exceeding 30% energy from sugar (or 40% for dairy-based products) are recommended to include this warning [15].

## Results

**Fig. 1:**
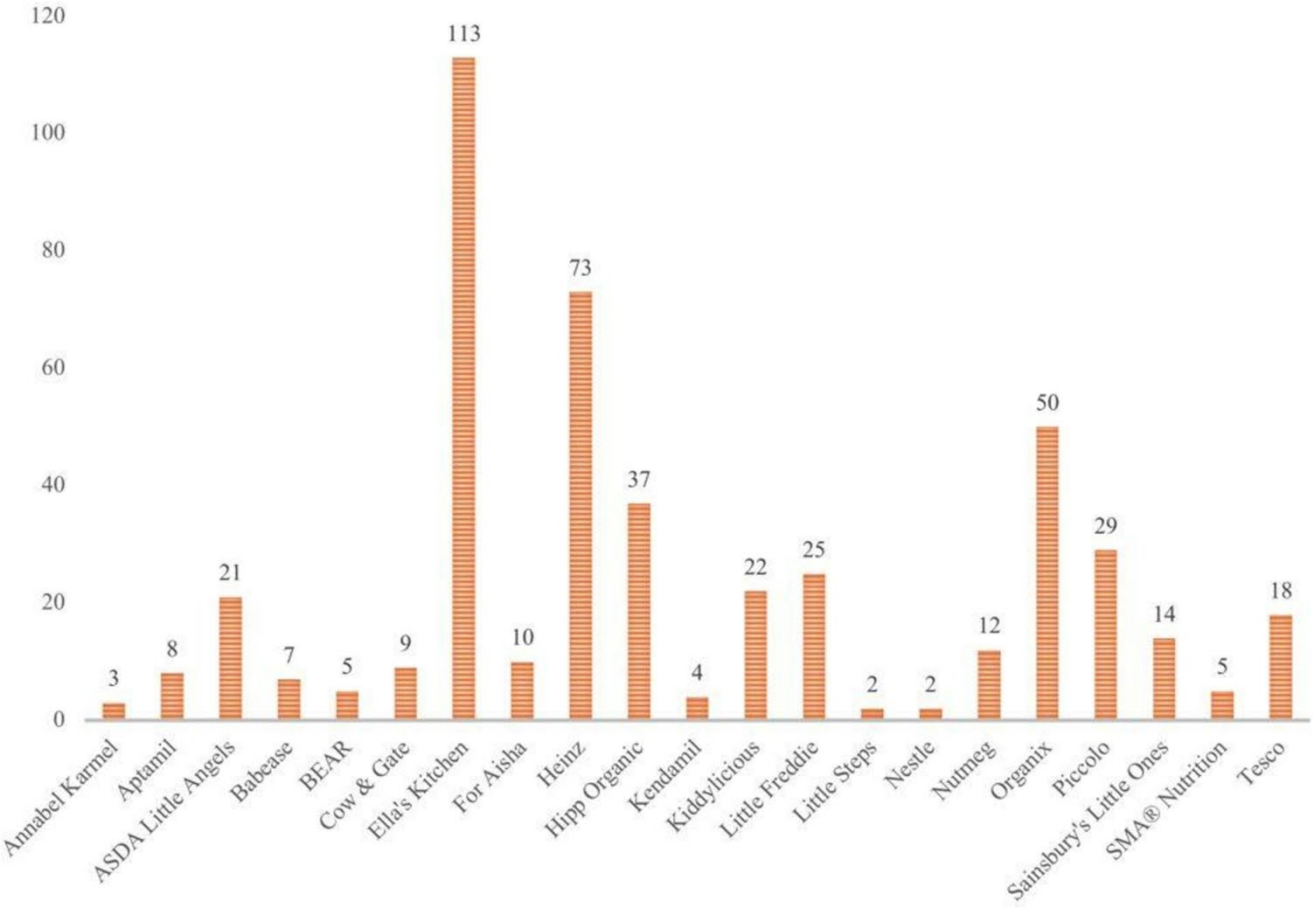
Product Brand Distribution

A total of 469 food products from 21 different brands were identified from the websites of four prominent supermarkets across the UK. Figure 1 illustrates the distribution of baby food products by brand in the UK. Notably, Ella’s Kitchen, Heinz, and Organix emerged as the top three brands.

As shown in the Table 1, most products fall under the meals category (38%, *n* = 178, followed by fruit and vegetables (30%, *n* = 139), and snacks (16%, *n* = 76). The confectionery and drinks category, which did not meet the NPPM criteria for marketing as baby food, accounted for 3% (*n* = 16) of all products. The majority of products are recommended for ages 6 to 9 months (55%), with a notable proportion recommended from 4 months (14%), and fewer aimed for 10 or 11 months (12%), and 12 to 24+ months (19%) (Table 1).

**Table 1:** Products category and lower recommendation age

	*n* (469)	%
Product Category		
Cereals	53	11
Dairy	6	1
Fruit & Vegetable	139	30
Meals	178	38
Snacks	76	16
Ingredients	1	<1
Confectionary	15	3
Drinks	1	<1
Lower age recommendation		
4 months	66	14
6–9 months	256	55
10–11 months	58	12
12–24 months	89	19

*n* number

### Nutritional composition

Table 2 presents the mean values (with 95% CI) for energy, protein, fat, sugar, and salt content per 100 g of products in different categories. Snacks have the highest energy content (416 kcal), while Fruit & Vegetable products have the lowest (64 kcal). Fat content was highest in snacks with a mean of 11.9 g per 100 g, followed by cereals with a mean of 5.5 g per 100 g. Dry cereals and Snacks have higher sugar contents and Fruit & Vegetable products are lowest in salt (Table 2).

**Table 2:** Nutritional composition per 100 g products by category

	Product Category					
	Dry cereals (*n* = 53)	Dairy (*n* = 6)	Fruit & Vegetable (*n* = 139)	Meals (*n* = 178)	Snacks (*n* = 76)	*p*
	Mean (95% CI)	Mean (95% CI)	Mean (95% CI)	Mean (95% CI)	Mean (95% CI)	
Energy (kcal)	391 (384, 397)*	74 (66, 82)	64 (61, 67)	69 (67, 71)	416 (402, 430)	<0.001
Protein (g)	12.1 (11.2, 13.0)	2.6 (2.3, 2.9)	1.3 (1.1, 1.4)	3.1 (2.9, 3.3)	7.5 (7.1, 7.9)	<0.001
Fat (g)	5.5 (4.3, 6.6)	2.3 (1.7, 2.9)	1.1 (0.9, 1.3)	2.2 (2.0, 2.3)	11.9 (10.7, 13.2)	<0.001
Sugar (g)	16.2 (12.8, 19.6)	4.4 (2.6, 6.2)	8.6 (8.0, 9.2)	2.5 (2.4, 2.8)	12.5 (9.3, 15.6)	<0.001
Salt (g)	0.21 (0.16, 0.27)	0.07 (0.06, 0.09)	0.03 (0.03, 0.04)	0.08 (0.07, 0.09)	0.18 (0.13, 0.23)	<0.001

*p* value obtained with the One-way Anova test, *CI* Confidence Intervals, Ingredient (small sample *n* = 1)

*For cereals, the energy content is presented based on the information for the dry product as specified on the label. Note that drink and confectionary items (should not be marketed based on NPPM guideline) were not evaluated

The energy per 100 g, protein, fat, and salt per 100 kcal, and sugar percent of energy (%) were evaluated based on the WHO NPPM requirements. 75% of products met the energy content standards, 98% adhered to fat content regulations, only 59% passed the total sugar content criteria, 91% to 94% met the protein content (as a percentage of total weight and g/ 100 kcal), and 86% complied with sodium content requirements. 19% of products failed because they contain added sugars (Supplementary Fig. 1).

Table 3 details the non-compliance rates for nutritional content across various subcategories within each main product category (cereals with/without milk, dairy, fruit & vegetables, meals, snacks, and ingredients). This encompasses parameters such as energy density, protein, fat, sugar, sodium, and fruit content. Each product has been deemed non-compliant for the overall assessment if it fails to meet any single criterion. High fail rates in specific categories, particularly Snacks (67%), Meals (64%), and Fruits and vegetables (46%) (Table 3), highlight areas where nutritional content necessitate improvement to align with the requisite standards. When categorized by age, almost 55% of products marketed for children under 12 months failed to meet criteria, while nearly 59% of products marketed for children aged 12 months and over also failed.

**Table 3:**
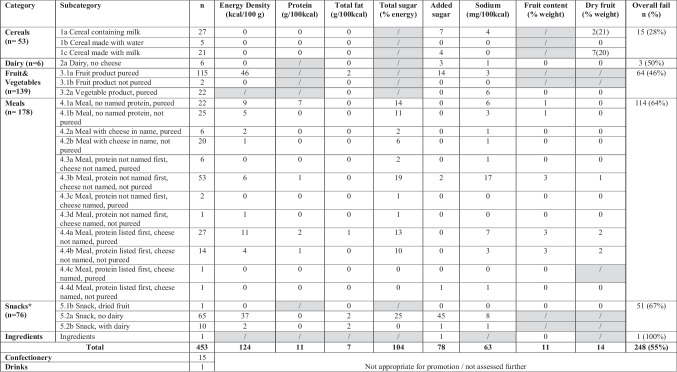
Nutritional content fails rates based on the NPPM criteria (Supplementary file Table 1)

*The energy density of snacks is evaluated as 50 kcal per serving. Rows highlighted in grey indicate that the nutrient contents do not need to be assessed based on the category

**Fig. 2:**
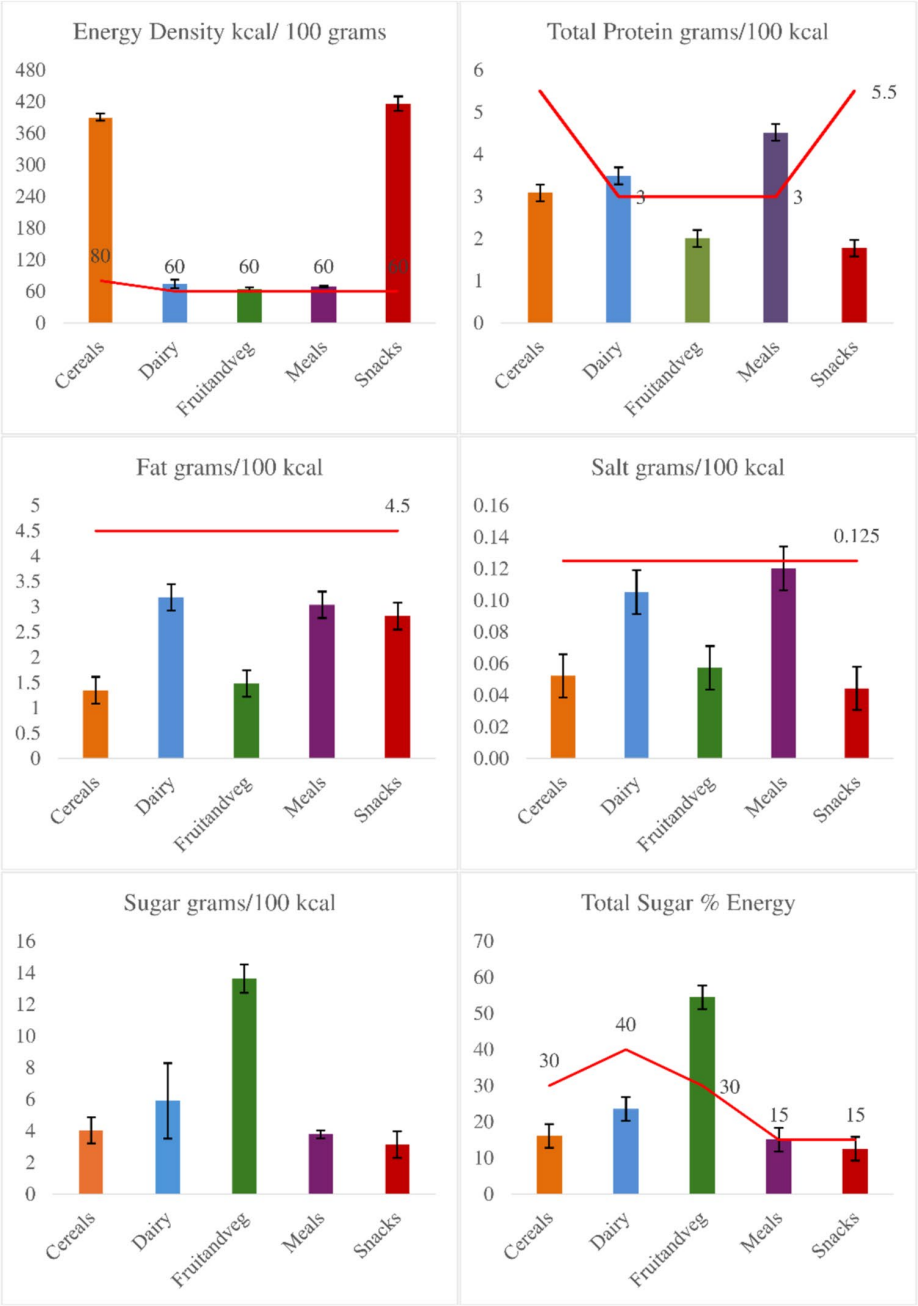
Nutritional contents of products by category. Notes: This figure depicts the nutritional content of the analysed products as assessed according to the NPPM criteria. Energy density, expressed in kilocalories (kcal) per 100 g of product. Total fat, protein, sugar and salt contents are presented grams per 100 kcal for each product on the figure. Notably, total sugar content is expressed as a percentage of total energy. According to NPPM criteria, products in the Confectionery and Drinks categories should not be marketed. Each graph within the figure displays the mean values with 95% confidence intervals (error bars) for these nutritional components. The red lines represent the criteria for nutrient contents as detailed in the methods (Supplementary file Table 1)

Additionally, the percentage of energy derived from sugar is displayed by category in Fig. 2. Fruits and vegetable category has the highest total sugar content (13.6 g with 0.9 g 95% CI) per 100 kcal and energy from sugar (55%) mean value compared the cereals, dairy, meals, and snacks.

### Promotional results

Table 4 evaluates whether products in each category meet specific promotional requirements, such as front-of-pack high sugar flags, age labels, claims, product name clarity, ingredient list clarity, preparation instructions, and instructions of breastfeeding.

**Table 4:**
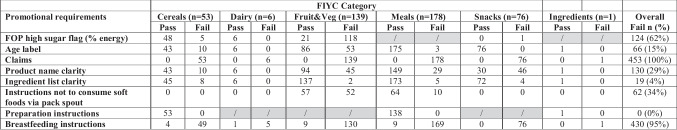
Promotional requirements by category

Rows highlighted in grey indicate that the promotional requirements do not need to be assessed based on the category

Analysis of product categorisation revealed products pass or fail from NPPM promotional requirements criteria. Notably, 62% of products in the cereals, dairy, fruits and vegetables, and snacks categories required a front-of-pack (FOP) high-sugar label due to their elevated sugar content (Table 4). The meals category exhibited the greatest non-compliance concerning breastfeeding instructions, with 169 products (95%) failing to meet this criterion. The cereals category exhibited the highest non-compliance rate (42%) regarding the misinformation ingredients requirements (typically this was where the ingredient list did not state the proportion of the largest ingredient, water/stock or fruit content). Products with a spout 84% of fruit and vegetable and %14 of meal category failed to include the guidance “children should not suck from the spout” (Table 4).

Overall, according to the NPPM requirements, none of the assessed products met the criteria for nutrient, health, marketing, and promotion claims. The assessment also revealed that 71% of products met the name clarity criteria, that is the product name reflected the main ingredients. However, only 63% of products met the preparation instructions criteria, with only 27% of spouted packaging labelled as not suitable for direct sucking. Regarding breastfeeding instructions, only 5% of products met the NPPM requirements, as detailed in the supplementary results (Supplementary fig. 1).

## Discussion

This study evaluated the compliance of UK-marketed complementary FIYC under 36 months with the NPPM. Of the 469 products assessed, 205 (45%) met the nutrient composition criteria, though none adhered to promotional requirements due to inappropriate claims on the labels.

Product availability varies geographically and is typically linked to neighbourhoods [21]. In this study, similar to the results of the Public Health England report [16], the most common product type was meals, followed by fruit and vegetable purées/smoothies, fruit desserts, snacks, and finger foods. The most common FIYCs in other countries are fruit and vegetable purees in Poland [22], Portugal [23], and Russia [24]; cereals in Turkey [25] and Indonesia; ready-to-eat finger foods and snacks in Cambodia; and pureed foods and meals in the Philippines [26].

Energy density is a critical aspect for FIYC ensuring that foods are adequate for growth and development. Vegetable purées do not have a minimum energy density requirement as they often have a naturally high-water content [21]. Different flavours and vegetables should still be offered during weaning (6 to 12 months). A maximum added water requirement is included for vegetable purées to ensure that they are not too low in energy. Fruit and vegetable purées were the product groups with the lowest energy density in this study, similar to Hutchinson et al. [27] and Santos et al. [23]. Low energy density can be a problem because babies’ and young children’s small stomachs limit their mealtime consumption [22]. The NPPM suggests a minimum energy density of at least 60 kcal/100 g for several product categories which represents a conservative benchmark against the energy density of breastmilk [15]. In the NPPM, dry cereals and starches must have a minimum energy density requirement of 80 kcal/ 100 g (as eaten) to ensure foods are more energy dense than milk and to align with existing EC requirements. Although most relevant products (75%) met the NPPM energy density levels, about half (40%) of the fruit purées and about a quarter (22%) of the meals were below the recommendations. In the NPPM pilot study, most products met the energy density standard (81% in the UK, 68% in Denmark, and 84% in Spain), although approximately half or less of the fruit purées and meals in the UK and Denmark met the energy recommendations [21].

Conversely, the consumption of snacks with high energy densities and low nutrient values can lead to excessive energy intake [28]. Snacks were the product category with the highest energy density in this study, and half (51%) of UK snacks exceeded the NPPM recommended energy per portion of 50 kcal (≤ 50 kcal per serving or portion) [15] compared to 72% in Turkey [25].

Most products did meet the protein requirements, similar to the results of the Polish study [22], except for some meals. High income countries, such as the UK with an already high protein intake even in early childhood the necessity of maintaining a high protein intake is nuanced. Whilst protein is essential for growth other nutrients from a range of food sources are also needed. Some FIYCs might have less protein than their homemade equivalents, resulting in an insufficient intake of essential nutrients [15, 29]. A high protein diet in infancy has been suggested as a risk factor for childhood overweight and obesity [30]. This raises the possibility of adding upper limits for protein to the NPPM.

Added sugars are widely used in various product categories, and a significant number of savoury meals contain pureed fruit, particularly in the UK [27]. According to the NPPM criteria, no products marketed for children under 36 months should contain added sugar [15]. However, this study found that 19% of products did contain added sugars. In a 27-country study by Grammatikaki et al., 39% of products contained sugar-added ingredients, with 10% listing added sugar, 14% free sugar, and 20% fruit purées and powders [31]. In the NPPM pilot study, 28% of UK, 21% of Danish, and 44% of Spanish products included added sugars [21]. Differences in sugar definitions and product types, such as the exclusion of sugars from vegetable purees, may account for these variations. Added sugars remain a concern, with NPPM defining them as monosaccharides and disaccharides added during processing, while free sugars include those naturally found in honey, syrups, and fruit concentrates. Liberated sugars, released during processing, also contribute to sweetness and rapid absorption [15].

In the new NPPM, the WHO has suggested that products with high sugar levels should not be marketed as appropriate for infants and young children. In addition, the NPPM recommends that products with more than 30% (cereals, fruit/vegetable purees), or 40% (dairy) calories from total sugars, should carry a front-of-pack flag on the label/packaging to indicate the presence of high sugar levels [15]. In this study, 46% of products exceeded FOP limits, and 41% surpassed total sugar thresholds. Nearly half of meals and one-third of snacks had excessive sugar (i.e., >15% of energy), with most fruit and vegetable purées exceeding 30% sugar content, making total sugar the most common cause of non-compliance. High sugar intake in infancy raises risks for dental caries and metabolic diseases [32].

Avoiding added salt in children’s foods is also crucial, as it affects taste preference and long-term health [33]. Although 86% of products met the salt limits, 22.5% of meal contained more salt than recommended. Compliance with sodium standards is higher in the US [34], New Zealand [35], and Portugal [23] but lower in Cambodia and Indonesia, where only one-third and half of the products, respectively, met the WHO NPM sodium standards [26]. Cambodia lacks sodium content standards for commercial FIYC products, while Indonesia’s sodium standard is more than twice the WHO NPM standard (50 mg/100 kcal) [26]. This highlights the role of compositional limits in managing nutrients of concern in commercial FIYC products.

In the draft NPPM applied across multiple countries, the percentage of items meeting all composition criteria ranged from 15% in Hungary to 42% in Estonia, with 31% of UK products meeting these criteria [21]. It should be noted that the draft NPPM differed slightly from the final version used here, as it did not include limits for dried or puréed fruit or energy density per serving for finger foods. In this study, 45% of UK products met the updated WHO NPPM criteria, compared to 29% in Turkey [25].

Promotion is considered inappropriate if it is misleading, confusing, or likely to lead to inappropriate use; for example, if it contains claims that idealize the products, compromise breastfeeding, or imply that they are better than family foods [36]. All products in this study displayed promotional claims on composition, nutrition, or health. Similarly, almost all products in four WHO European countries (95–100%) [37] and seven Southeast Asian countries (98.6%) [38], carried some type of statement on composition, nutrition, or health-related promotions.

The WHO recommends exclusive breastfeeding until six months, followed by continued breastfeeding alongside complementary foods up to two years or beyond. This guideline is also supported by UK public health authorities, which advise against introducing complementary foods before six months [40, 41]. Breastfeeding duration is shorter in high-income countries than those that are resource poor. Despite evidence of protection against childhood conditions and later life ill health for the mother. Breastfeeding up to two years and beyond supports the health and neurobiological development of an infant and a young child [40]. Although UK market surveys show a decrease in products marketed for infants under six months (from 43% in 2013 to 23% in 2019), the range of products targeting this age group has increased from 178 to 201 types [17]. In present study, 14% of products were still marketed as suitable for infants under six months, highlighting a need for regulatory action.

The high non-compliance rates, particularly regarding “claims and breastfeeding support,” highlight the need for stricter promotional regulations to avoid conflicts with public health messages and maintain caregivers’ trust in baby foods. This study found that only 45% of UK FIYC products met nutrient composition criteria, with high sugar content being the main area of non-compliance, and none met NPPM promotional standards. These findings underscore the importance of comprehensive labelling standards and regulatory updates to reduce misleading claims and added sugars, promoting healthier dietary patterns for infants and young children.

This study has several limitations. Firstly, it relied on product label information from supermarket websites, which did not always fully meet NPPM standards (e.g., fruit content, added water, protein, age recommendations). Additionally, the lack of data on the proportion of commercial versus home-prepared foods consumed by UK infants and young children may limit the generalisability of the findings. Secondly, the analysis relied on manufacturer-reported nutrient content rather than independent laboratory testing.

## Conclusion

The NPPM has been a useful tool to identify improvements in the nutritional quality and promotion of UK baby foods. Under half of the products reviewed met the nutritional standards showing the need to reformulate. None of the products met all the promotion criteria and few products supported breastfeeding statements. However, the NPPM could be further developed in future, potentially adding upper limits to protein in specific product categories and considering a wider range of important nutrients. This will further support international comparisons where nutrients such as iron may be a consideration. As a first step to highlight needs for commercial products to be improved the NPPM set the standard, and it has been used by other countries as a basis for recommendations.

## Supplementary information

ESM 1 (DOCX 540 kb)

## Data Availability

https://archive.researchdata.leeds.ac.uk/1185/

## Abbreviations

NPPM: Nutrient and Promotion Profile Model
FIYC: Foods for infants and young children
FOP: Front-of-pack
