# Supplementary material for: Evaluating alignment of UK commercial baby food products with the WHO nutrient and promotion profile model
Source: Eur J Pediatr. 2025 Jan 11;184(2):128. doi: 10.1007/s00431-025-05971-7 (PMC11724783; doi:10.1007/s00431-025-05971-7)
Supplement: Supplementary file 1 — (DOCX 540 kb) [file 431_2025_5971_MOESM1_ESM.docx]

**Table 1. NPPM Part A: content and front-of-pack labelling**

| **Product group** | **Code** | **Sub-category description** | **Details and examples** | **Energy density (kcal/100 g)** | **Sodium**  **(mg/100 kcal)** | **Total sugar (%E)** | **Added free sugars** | **Total Protein (g/100 kcal)** | **Total fat (g/100 kcal)** | **Fruit content (% weight)** | **Age label (months)** | **Front-of-pack high sugar flag (% E)** |  |
| --- | --- | --- | --- | --- | --- | --- | --- | --- | --- | --- | --- | --- | --- |
| **Dry cereals and starches** | 1 | Dry or powdered cereal/starch to be eaten or cooked with milk or water | To be prepared with milk (or equivalent non-sweet liquid) or water (or protein-free liquid) e.g. Instant porridge, muesli, baby rice, dry pasta. Includes plain fresh pasta etc. Excludes crackers/rusks etc. (Cat. 5.2) | ≥ 80 as eaten | ≤ 50 as eaten | / | None | ≤5.5g (if contains milk) | ≤4.5 g or ≤3.3 g (if to be eaten with m ilk) | ≤ 10% dry weight | 6-36 | ≥30% |  |
| **Dairy foods** | 2 | Dairy-based foods, desserts and cereals | The largest ingredient is dairy and fruit ≤5% e.g. Porridge, Rice pudding, Yogurt, Fromage frais, Custard. [If fruit content >5% use Cat.3.1] | ≥ 60 | ≤ 50 (100 if named cheese) | / | None | / | ≤4.5 g | ≤ 5% (max 2% dry) | 6-36 | ≥40% |  |
| **Fruit & vegetable purees/ smoothies and fruit desserts** | 3.1 | Fruit-containing product, including breakfast/ dairy | Any product containing >5% fruit^1^ (except dry cereals, low fruit dairy, or snacks) e.g. Apple puree, Fruit and yogurt, Fruit custard, Porridge with >5% fruit | ≥ 60 | ≤ 50 | / | None | / | ≤4.5 g | / | 6-36  (6–12 for purée) | ≥30% |  |
|  | 3.2 | Vegetable only product | Single or mixed vegetables or legumes e.g. Spinach & pea puree, Mashed potato & carrot. Excludes foods containing added starch/fat/dairy (Cat. 4.1] | ≤ 25% added water | ≤ 50 | / | None | / | ≤4.5 g | None | 6-36  (6–12 for purée) | ≥30% |  |
| **Savoury meals and meal-components** | 4.1 | Food WITHOUT protein or cheese named | Vegetables/legumes and/or cereals/starches. May contain a protein source, dairy or fats e.g. Vegetable rice, Lasagne, Pesto sauce for pasta | ≥ 60 | ≤ 50 | ≤ 15% | None | ≥3 g | ≤4.5 g | ≤ 5% (max 2% dry) | 6-36  (6–12 for purée) | / |  |
|  | 4.2 | Food WITH CHEESE named but no protein | Cheese and no other proteins are in the product name e.g. Cheese pasta, Tomato & Mozzarella pasta sauce | ≥ 60 | ≤ 100 | ≤ 15% | None | ≥3 g | ≤ 6 g | ≤ 5% (max 2% dry) | 6-36  (6–12 for purée) | / |  |
|  | 4.3 | Food with protein source NOT named first | Protein source is not the first named food, e.g. Pea & lamb curry, Tomato & Beef sauce for pasta | ≥ 60 | ≤ 50 (100 if named cheese) | ≤ 15% | None | ≥3 g | ≤ 4.5 g | ≤ 5% (max 2% dry) | 6-36  (6–12 for purée) | / |  |
|  | 4.4 | Food with protein source named FIRST | e.g. Rabbit & potato, Beef soup, tasty chicken risotto, Chicken & cheese pasta, Beef sauce for pasta | ≥ 60 | ≤ 50 (100 if named cheese) | ≤ 15% | None | ≥4 g | ≤ 6 g | ≤ 5% (max 2% dry) | 6-36  (6–12 for purée) | / |  |
|  | 4.5 | Protein source is ONLY named food | Pureed cooked meat. May contain a small quantity of grain/starch not in product name e.g. ‘Rabbit’ or ‘Lamb’ with some added rice flour or cornstarch | ≥ 60 | ≤ 50 | ≤ 15% | None | ≥7 g | ≤ 6 g | ≤ 5% (max 2% dry) | 6-36  (6–12 for purée) | / |  |
| **Snacks and finger foods** | 5.1 | Fruit | Fresh fruit or whole dry fruits or pieces e.g. plain dry apple slices or raisins. Excludes pulverised/pureed dry fruits (Cat. 7) | ≤ 50kcal per serve | ≤ 50 | / | None | / | ≤ 4.5 g | 100 % | 6-36 | ≥30% (dry fruit only) |  |
|  | 5.2 | Dry or semi-dry snacks and finger foods | Any grain, starch, pulse/lentil or root vegetable snack such as cracker, bread, biscuit, pastry, cake or pancake etc. Includes rusks, crackers and biscuits to be eaten dry or pulverised with liquid | ≤ 50kcal per serve | ≤ 50 | ≤ 15% | None | ≤5.5g (if biscuit and contains milk) | ≤ 4.5 g | / | 6-36 | / |  |
| **Ingredients** | 6 | Ingredients | Ingredients for cooking or adding to food in small quantities e.g. olive oil, stock cubes | / | ≤ 50 | / | None | / | / | None | 6-36 | / |  |
| **Confectionery** | 7 | Confectionery | Chocolates, sweets, liquorice, marzipan, fruit chews^3^ etc. | Not appropriate for promotion | | | | | | | | | |
| **Drinks** | 8 | Drinks | Fruit juice and other sweetened or flavoured drinks.^4^ Excludes 100% fruit/vegetable puree, breast-milk substitutes or unsweetened milk/milk alternatives | Not appropriate for promotion | | | | | | | | | |

^1^ Notes on fruit: Tomatoes, avocadoes and coconut are not classed as fruits for this purpose.

^2^ Note that the front of pack and legal product names and order of foods may differ. Follow the front-of-pack names for product categorisation where possible.

^3^ Fruit chews include any dried and processed fruit products such as fruit gums, bars or fruit strips/leathers/roll-ups (i.e. a dense chewy food made from fruit juice or pulped and dehydrated/dried fruit)

^4^ Includes any drinkable product containing crushed, blended, pulped or puréed fruit/vegetable, fruit/vegetable juice and/or water, with or without added free sugar or sweetening agents. Including 100% juices, reconstituted juice from concentrate, smoothies with added juice or water, drinks made from cordials, energy drinks, ices, and soft drinks.

^5^ Note on Added Sugars:

Added Sugars: Defined as all monosaccharides and disaccharides added to foods and beverages by the manufacturer, cook, or consumer during processing or preparation.

Free Sugars: Include monosaccharides (e.g., glucose, fructose) and disaccharides (e.g., sucrose) added by the manufacturer, cook, or consumer, as well as sugars naturally present in honey, syrups, fruit juices, and fruit concentrates.

Liberated Sugars: These are sugars released from plant cell walls during processing, such as heat-treatment, maceration, or puréeing, functioning similarly to free sugars by enhancing sweetness and increasing the rate of blood sugar absorption. For instance, fruit purée is high in liberated sugars, contributing to a sweeter taste and a rapid rise in blood sugar when consumed alone or as an ingredient.

Banned Free Sugars and Sweeteners: Include: i. All monosaccharides and disaccharides (from sources like fruits, sugarcane, palms, and root vegetables). ii. All syrups, nectars, and honey (e.g., molasses, agave, maple syrup, blossom nectar, malted barley syrup). iii. Fruit juices or concentrated/powdered fruit juice, excluding lemon or lime juice (e.g., pear juice, concentrated apple juice, powdered mango juice). iv. All non-sugar sweeteners (e.g., saccharin, acesulfame, aspartame, sucralose, stevia).

**Table 2. NPPM Part B: promotional messages (packets, labelling and marketing)**

| **Promotional requirement** | **Details and examples** |
| --- | --- |
| **No compositional, nutritional, health or marketing claims** | No compositional, nutritional, health or marketing claims are permitted on packs or related marketing materials (promotional communications, websites, etc.). Refer to Table 3 for examples of non-permitted claims.  Note the following composition statements **are permitted**: i. statements relating to common allergens (such as containing or being “free from...[gluten, dairy/lactose, or nuts]”etc.) ii. statements relating to religious or cultural requirements (such as “meat-free”, “vegetarian”, “contains meat”, “Kosher”, “Halal”, etc.)  iii. descriptive words may be used *within* the ingredient list (such as “organic carrots” and “wholegrain wheat flour”) |
| **Product name clarity^1^** | The front-of-pack product name and legal product name must:  i. clearly represents or name the main or largest ingredients, where appropriate, except when the largest ingredient is implied in the name (such as milk in porridge or rice in risotto);  ii. list ingredients in an appropriate order(to indicate decreasing proportional content); and  iii. indicate when fruit or vegetables (single or in combination) comprise the majority of the product by weight. Note that fruit or vegetables are the largest ingredient if the sum of all fruits or vegetables is the largest ingredient, and the front-of-pack name must indicate this (see example (iii) in footnote 1)  Note that all ingredients do not need to be listed in the product name |
| **Ingredient list missing clarity** | The ingredient list must clearly indicate the proportion (%) of:  i. the largest single ingredient (including water/stock, except when used for rehydration of legumes/grains etc.) ii. The amount of added water/stock(except when used for rehydration of legumes/grains etc.)  iii. the total or individual proportions of fresh or dried fruit  iv. the amount of fish, poultry, meat or other traditional source of protein |
| **Instructions not to consume soft foods via spout (if has spout)** | Ready-to-eat puréed foods sold in packs with a spout must include a clear statement to discourage caregivers from allowing infants and young children to suck the food directly via the spout, such as: “Infants and young children should not be allowed to suck directly from the pouch/container” |
| **Suitable preparation instructions** | Preparation instructions for dry cereals/starches, ingredients and meal components must state that the liquid used to reconstitute the product, or accompanying foods served, should have no added sodium or free sugar (including fruit juice) |
| **Promotion and protection breastfeeding** | In relation to breast feeding:  i. no cross-promotions are permitted between products that function as breastmilk substitutes, and commercially available complementary foods marketed as suitable for infants and young children > 6 months;  ii. all products must include a statement on the importance of continued breastfeeding for up to two years or beyond and the importance of not introducing complementary feeding before 6 months of age;  iii. no products should include any image, text or other representation that is likely to undermine or discourage breastfeeding, or that makes a comparison to breastmilk or that suggests that the product is nearly equivalent or superior to breastmilk;  iv. all products must state the suitable age of introduction (≥ 6 months);  v. no products should include any image, text or other representation that might suggest use for infants under the age of 6 months (including references to milestones and stages); and  vi. no product should convey an endorsement or anything that may be construed as an endorsement by a professional or other body, unless this has been specifically approved by relevant national, regional, or international regulatory authorities. |

^1^ Improved product name examples:

i. 30%apple,20%sweetpotatoand10%spinach:

before: “spinach and sweet potato”; after: “apple, sweet potato and spinach”

name ingredients in an appropriate order and indicate that apple is the main ingredient.

ii. 35% carrot, 30% parsnip, 20% potato and 15% chicken:

before: “chicken and vegetable meal”; after: “root vegetable and chicken dinner” or “carrot and potato mash with chicken”

Indicate that vegetables are the largest ingredient.

iii. 30% pear, 20% apple, 20% rice/oats/dairy and 10% strawberry:

before: “baby rice/porridge/yogurt with strawberry”; after: “pear and apple porridge/rice/yogurt with strawberry” or “fruity rice/porridge/yogurt”

Indicate that fruit is the largest ingredient, strawberry is not the primary fruit and rice/oats/yogurt is not the largest ingredient.

**
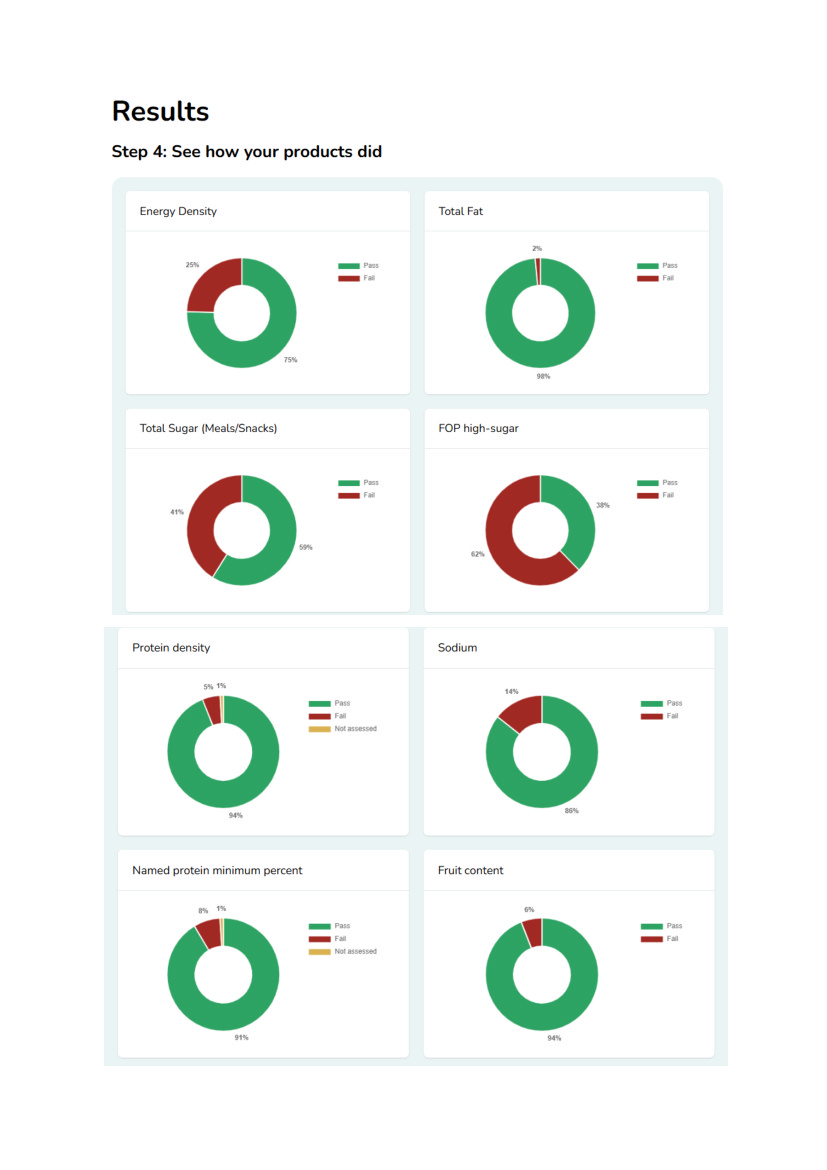

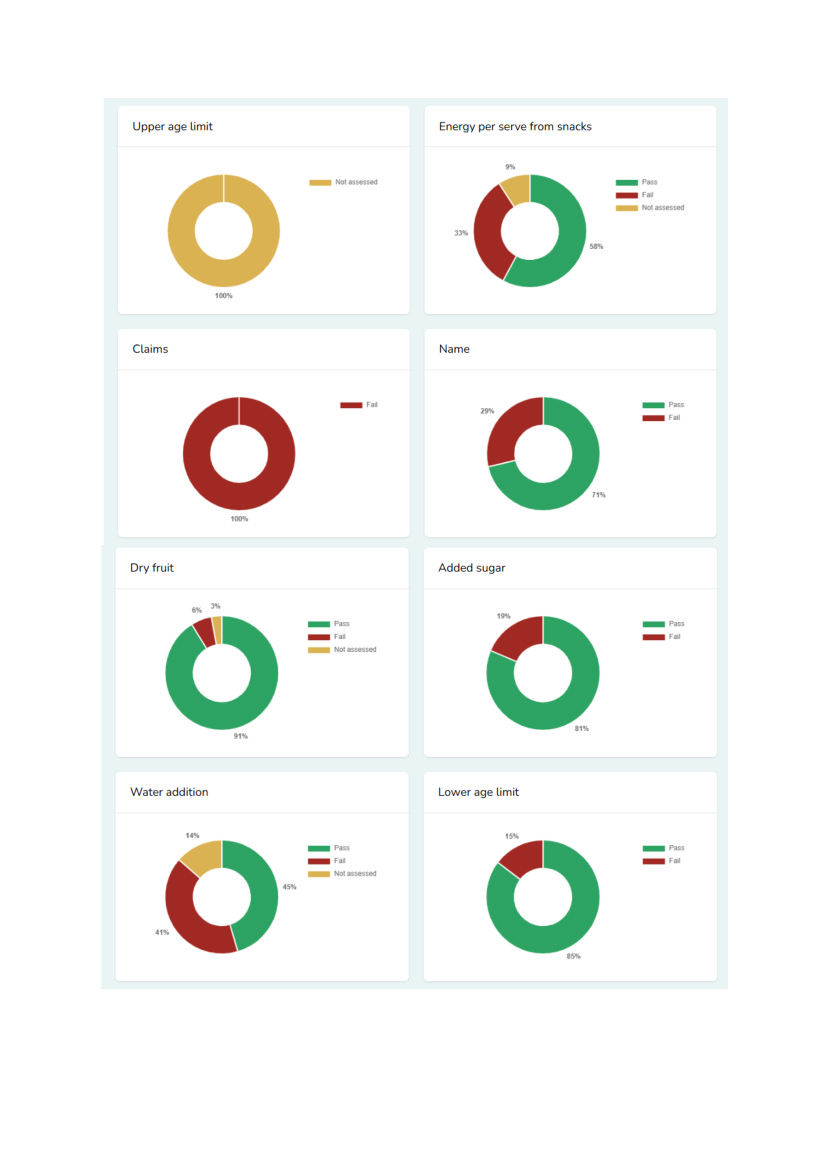

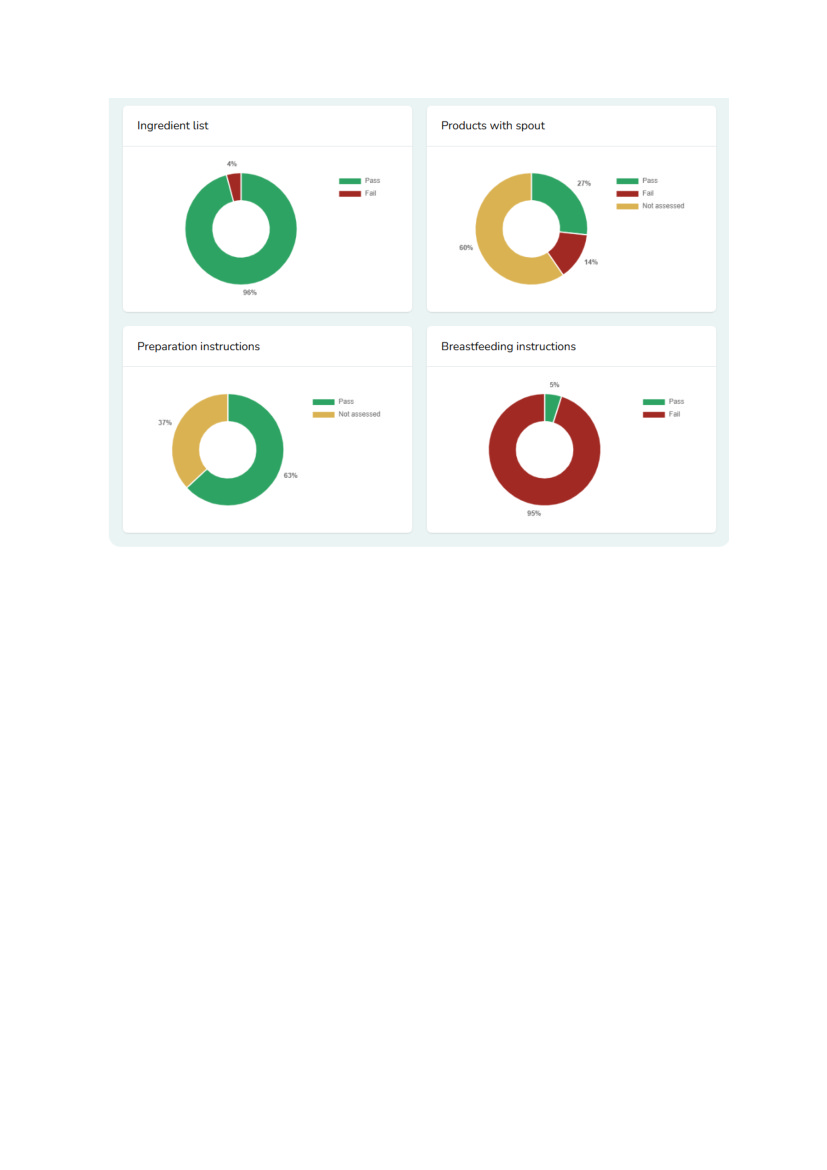
**

**Figure 1.** UK FIYC Data Analysis: From NPPM Website (screen shots)
